# Predicting protein–protein interactions in microbes associated with cardiovascular diseases using deep denoising autoencoders and evolutionary information

**DOI:** 10.3389/fphar.2025.1565860

**Published:** 2025-03-11

**Authors:** Senyu Zhou, Jian Luo, Mei Tang, Chaojun Li, Yang Li, Wenhua He

**Affiliations:** ^1^ Cardiovascular Department, The Fourth Hospital of Changsha (Integrated Traditional Chinese and Western Medicine Hospital of Changsha, Changsha Hospital of Hunan Normal University), Changsha, China; ^2^ School of Computer Science and Information Engineering, Hefei University of Technology, Hefei, China

**Keywords:** protein–protein interactions, cardiovascular disease, deep denoising autoencoder, CatBoost, evolutionary information

## Abstract

**Introduction:**

Protein–protein interactions (PPIs) are critical for understanding the molecular mechanisms underlying various biological processes, particularly in microbes associated with cardiovascular disease. Traditional experimental methods for detecting PPIs are often time-consuming and costly, leading to an urgent need for reliable computational approaches.

**Methods:**

In this study, we present a novel model, the deep denoising autoencoder for protein–protein interaction (DAEPPI), which leverages the denoising autoencoder and the CatBoost algorithm to predict PPIs from the evolutionary information of protein sequences.

**Results:**

Our extensive experiments demonstrate the effectiveness of the DAEPPI model, achieving average prediction accuracies of 97.85% and 98.49% on yeast and human datasets, respectively. Comparative analyses with existing effective methods further validate the robustness and reliability of our model in predicting PPIs.

**Discussion:**

Additionally, we explore the application of DAEPPI in the context of cardiovascular disease, showcasing its potential to uncover significant interactions that could contribute to the understanding of disease mechanisms. Our findings indicate that DAEPPI is a powerful tool for advancing research in proteomics and could play a pivotal role in the identification of novel therapeutic targets in cardiovascular disease.

## 1 Introduction

Cardiovascular diseases (CVDs) are a major global health challenge, accounting for a significant proportion of morbidity and mortality. These diseases encompass a range of conditions affecting the heart and blood vessels, including coronary artery disease, heart failure, and stroke (Tada et al., 2022; Gaidai et al., 2023). The multifactorial nature of CVDs is influenced by various elements, such as genetic predispositions, lifestyle factors, and environmental exposures (Dale et al., 2023; Ojeda-Granados et al., 2024). At the molecular level, proteins play a pivotal role in the pathophysiology of CVDs as they are integral to processes such as inflammation, lipid metabolism, and vascular function (Frąk et al., 2022). Understanding the intricate relationships between these proteins and their interactions is crucial for unraveling the mechanisms underlying cardiovascular pathologies. Thus, the study of protein–protein interactions (PPIs) holds significant promise for advancing our knowledge of CVDs and identifying potential therapeutic targets (Greenblatt et al., 2024).

The importance of PPIs has garnered increasing attention within the scientific community, positioning them as a focal point in biological research. Experimental methods for large-scale PPI identification, such as yeast two-hybrid screening (Wong et al., 2017) and affinity purification coupled with mass spectrometry (Zhong et al., 2023), have been developed to explore these interactions. However, these biological techniques often suffer from limitations, including high costs, time demands, and the potential for false-positive or false-negative results. Consequently, there has been a growing need for effective computational methods to predict PPIs, offering a complementary approach that can enhance the accuracy and efficiency of PPI identification (Xian and Wang, 2024).

Numerous computational strategies have emerged (Guo et al., 2024; Wei et al., 2024), leveraging a wide variety of data types to predict PPIs. For example, You et al. (2013) proposed a PCA-EELM model, which extracts features from protein sequences by autocovariance scores, conjoint triad scores, local descriptor scores, and autocorrelation scores and then combines PCA and ensemble ELM to predict PPIs. Li et al. (2020) adopted a weighted ELM integrated with a scale-invariant feature transform algorithm for accurate prediction. Zhang et al. (2019) implemented an ensemble deep neural network model that leveraged three representative protein feature extraction descriptors combined with a separate deep neural network, achieving high accuracy in predicting PPIs. Hashemifar et al. (2018) presented a sequence-based deep learning framework, DPPI, which combines convolutional and random projection modules in order to enhance the prediction accuracy. Halder et al. (2020) developed a new method called JUPPI, which takes the sequence, GO, and structural domain information of proteins as input features; constructs a high-quality negative PPI dataset by the proposed three-stage filtering strategy; and combines it with a random forest classifier to achieve remarkable results in detecting PPIs of the human proteome. Ma et al. (2024) employed CollaPPI, a collaborative learning framework with information sharing, to predict PPIs, which showed the cross-domain knowledge complementarity of collaborative learning methods in this task. Despite the significant advancements in these computational methods, challenges remain in improving the efficiency and accuracy of PPI predictions. As the volume of protein sequence data continues to increase exponentially, there is an increasing need for effective models that can operate solely on sequence information. This emphasizes the importance of ongoing research to refine and develop innovative computational techniques for PPI prediction, ensuring they can meet the demands of the scientific community.

In this study, we introduce a novel computational model, the deep denoising autoencoder for protein–protein interaction (DAEPPI), which is designed to predict PPIs by utilizing evolutionary information extracted from amino acid sequences. We provide a detailed breakdown of the model architecture and its components, highlighting how each aspect contributes to its predictive capability. Our extensive experimental validation showcases the model’s effectiveness in predicting PPIs, with impressive performance results on both yeast and human datasets. Furthermore, we present comparative experiments that underscore the reliability of the DAEPPI model. Notably, we explore its application in the context of cardiovascular diseases, demonstrating its potential implications for understanding protein interactions that contribute to these conditions. In conclusion, through this research, we aim to further elucidate the role of PPIs in cardiovascular disease mechanisms and their significance for future therapeutic strategies.

## 2 Materials and methods

### 2.1 Data sources

In this study, we constructed two comprehensive PPI datasets from distinct organisms: *Saccharomyces cerevisiae* (yeast) and *Homo sapiens* (human). The yeast dataset was sourced from the Database of Interacting Proteins (DIP) (Salwinski et al., 2004), where we carefully filtered out protein pairs containing fewer than 50 residues or exhibiting over 40% sequence identity. This rigorous selection process resulted in a positive dataset comprising 5,594 interacting protein pairs, complemented by an equal number of non-interacting pairs derived from proteins with differing subcellular localizations, culminating in a total of 11,188 protein pairs.

For the human dataset, we utilized the Human Protein Reference Database (HPRD). After excluding pairs with more than 25% sequence identity, we identified 3,899 verified interacting pairs among 2,502 unique human proteins. Additionally, we created a negative dataset consisting of 4,262 pairs from 661 distinct proteins, ensuring that these non-interacting proteins were sourced from various subcellular compartments (You et al., 2014). Ultimately, the human dataset comprised a total of 8,161 protein pairs. Together, these datasets provide a robust foundation for evaluating our DAEPPI method in predicting protein interactions across different biological contexts.

### 2.2 Position-specific scoring matrix

The position-specific scoring matrix (PSSM) (Liu and Steinegger, 2023) is a powerful tool widely used for identifying distantly homologous proteins, as well as for sequence-level studies such as sequence alignment and conservation analysis. In our research, we employed PSSM to enhance the prediction of PPIs. Each protein sequence was converted into a PSSM using the position-specific iterated basic local alignment search tool (PSI-BLAST) (Altschul et al., 1997; Jin et al., 2021). The PSSM is structured as an L×20 matrix, where *L* represents the length of the protein sequence, and the 20 columns correspond to the standard amino acids. The PSSM of a protein sequence is defined as follows:

$$P_{PSSM}=\left[\begin{array}{cccc} x_{1,1} & x_{1,2} & \cdots & x_{1,20}\\ x_{2,1} & x_{2,2} & \cdots & x_{2,20}\\ \vdots & \vdots & \vdots & \vdots \\ x_{L,1} & x_{L,2} & \cdots & x_{L,20} \end{array}\right],$$ (1)

where each element $x_{i,j}$ in the matrix provides a score for the *j*-th amino acid at the *i*-th position, which is calculated based on the frequency of amino acids at that position and their evolutionary substitutions. To construct this matrix effectively, we configured PSI-BLAST with an e-value threshold of 0.001 and conducted three iterations to ensure a comprehensive collection of homologous sequences. By utilizing PSSM, we can not only capture the conserved patterns within protein sequences but also utilize the evolutionary information encoded within protein sequences to improve the accuracy of the PPI prediction task.

### 2.3 Deep denoising autoencoder

After obtaining the PSSM of a protein sequence, we need to perform feature extraction on it to represent each protein effectively. However, since the length of protein sequences is not fixed, the size of the constructed PSSM matrices will be different, which makes it impossible to directly input them into the deep learning model. To solve this problem, we adopt a uniform-sized PSSM matrix ${\hat{P}}_{PSSM}$ , i.e., by introducing transposed PSSMs and thus transforming each PSSM into a 20×20 matrix of fixed size. This process can be formulated as follows:

$${\hat{P}}_{PSSM}=P_{PSSM}^{T}\times P_{PSSM}$$ (2)

Next, in this study, we employed the denoising autoencoder (DAE) algorithm (Vincent et al., 2008; Peng et al., 2023) to extract deeper feature representations of protein sequences from the generated ${\hat{P}}_{PSSM}$ matrix.

From a mathematical point of view, the process of encoding can be defined as follows:

$$h=f\left(Wx+b\right)$$ (3)

Here, f denotes the non-linear activation function, x denotes the original input, W denotes the weight of the encoder, and b denotes the bias of the encoder. Similarly, the output of the decoder can be defined as follows:

$$\hat{x}=f\left(\hat{W}h+\hat{b}\right)$$ (4)

where the encoder compresses the input x into a potential space h corresponding to the feature we want to learn, and then the latent structure is reinstated in the decoded output $\hat{x}$ via the decoder. Here, $\hat{b}$ denotes the bias of the decoder, and $\hat{W}$ denotes the weight of the decoder. For an original training dataset ${\left\{x_{i}\right\}}_{i=1}^{N}$ , the entire training phase of the encoder–decoder framework can be described as follows:

$${\hat{x}}_{i}=f\left(\hat{W}\left(Wx_{i}+b\right)+\hat{b}\right)$$ (5)

The DAE model is particularly effective for this purpose as it enhances the robustness of the feature representation by reconstructing inputs that have been intentionally corrupted with noise. This process also involves two primary phases: encoding and decoding. Formally, the DAE first adds noise to the original data x to form the corrupted input $\tilde{x}$ . This process can be realized using a stochastic mapping $\tilde{x}∼q_{D}\left(\tilde{x}|x\right)$ , which can be expressed as follows:

$$\tilde{x}=x+\alpha$$ (6)

Then, as in the case of the autoencoder, the corrupted input $\tilde{x}$ is mapped to the hidden representation y through the encoder $f_{\vartheta }$ , which is denoted as follows:

$$y=f_{\vartheta }\left(\tilde{x}\right)=s\left(W\tilde{x}+b\right)$$ (7)

Consequently, we use the decoder $g_{\vartheta^{\prime }}$ in the decoding process to reconstruct the uncorrupted z by mapping to the learned y , which is denoted as follows:

$$z=g_{\vartheta^{\prime }}\left(y\right)=s\left(W^{\prime }y+b^{\prime }\right)$$ (8)

where s is the sigmoid nonlinear activation function of the decoder, denoted as follows:

$$s\left(x\right)=\frac{1}{1+e^{-x}}$$ (9)

After obtaining the uncorrupted input z , the DAE determines the reconstruction data loss and continuously optimizes the model parameters by calculating the minimized squared error between the original input x and z , as follows:

$$L\left(x,z\right)=∥x-z{∥}^{2}$$ (10)

$$\theta^{*},\theta^{\prime *}=\mathrm{argmin}\frac{1}{n}\sum_{i=1}^{n}L\left(x^{\left(i\right)},z^{\left(i\right)}\right)$$ (11)

Ultimately, by taking the PSSM of protein sequences as input and then performing encoding–decoding operations, DAE ensures that the learned features are not only lower-dimensional but also more representative of the underlying biological information. Thus, we can enhance our DAEPPI computational model to fully utilize the evolutionary insights embedded within the PSSM.

### 2.4 Categorical boosting (CatBoost)

In our classification task for predicting protein interactions, we used the CatBoost algorithm, a powerful gradient-boosting framework that excels in handling categorical features. CatBoost builds on the traditional gradient boosting decision tree (GBDT) method, employing oblivious trees as base learners to enhance the accuracy and generalization while effectively addressing issues such as gradient bias and prediction shift (Dorogush et al., 2018; Zhang et al., 2023). CatBoost handles categorical features well and allows training on the entire original dataset. The CatBoost algorithm computes the categorical features by first randomly permuting the given raw data into $\sigma =\left(\sigma_{1},\sigma_{2},\ldots ,\sigma_{n}\right)$ and then substituting it with the following numerical features:

$${\hat{x}}_{k}^{i}=\frac{\sum_{j=1}^{p-1}\left[x_{\sigma_{j},k}=x_{\sigma_{p},k}\right]\cdot Y_{\sigma_{j}}+\alpha \cdot p}{\sum_{j=1}^{p-1}\left[x_{\sigma_{j},k}=x_{\sigma_{p},k}\right]+\alpha }$$ (12)

where p denotes the *a priori* value, α>0 denotes the weight of the *a priori* value, $x_{\sigma_{j},k}$ represents the category features, and $Y_{\sigma_{j}}$ represents the label of the corresponding feature.

To classify the features extracted from the DAE, we first prepared our dataset by combining the DAE-derived feature representations with the corresponding labels. These features, which capture the essential characteristics of the proteins, were then input into the CatBoost classifier. The model was trained several times to learn the relationship between features and their labels to further optimize the hyperparameters of the task. One of the significant advantages of CatBoost is its ability to process categorical variables directly during the training phase, which eliminates the need for extensive preprocessing. By employing target statistics and ordered boosting, CatBoost minimizes information loss, while enriching the feature space, allowing for a more nuanced understanding of the data. This robust classification strategy enabled us to accurately predict protein interactions based on the rich feature representations learned from the DAE, ultimately improving the reliability of our PPI predictions.

### 2.5 Evaluation measures

To verify the validity of the constructed DAEPPI model, we used four classical evaluation criteria for measurement (Liang et al., 2025). They are accuracy (ACC), precision (PE), sensitivity (SN), and Matthews correlation coefficient (MCC), which are defined as follows:

$$ACC=\frac{TN+TP}{TN+TP+FN+FP}$$ (13)

$$PE=\frac{TP}{FP+TP}$$ (14)

$$SN=\frac{TP}{TP+FN}$$ (15)

$$MCC=\frac{\left(TP\times TN\right)-\left(FP\times FN\right)}{\sqrt{\left(TP+FP\right)\times \left(TN+FN\right)\times \left(TN+FP\right)\times \left(TP+FN\right)}}$$ (16)

**FIGURE 1:**
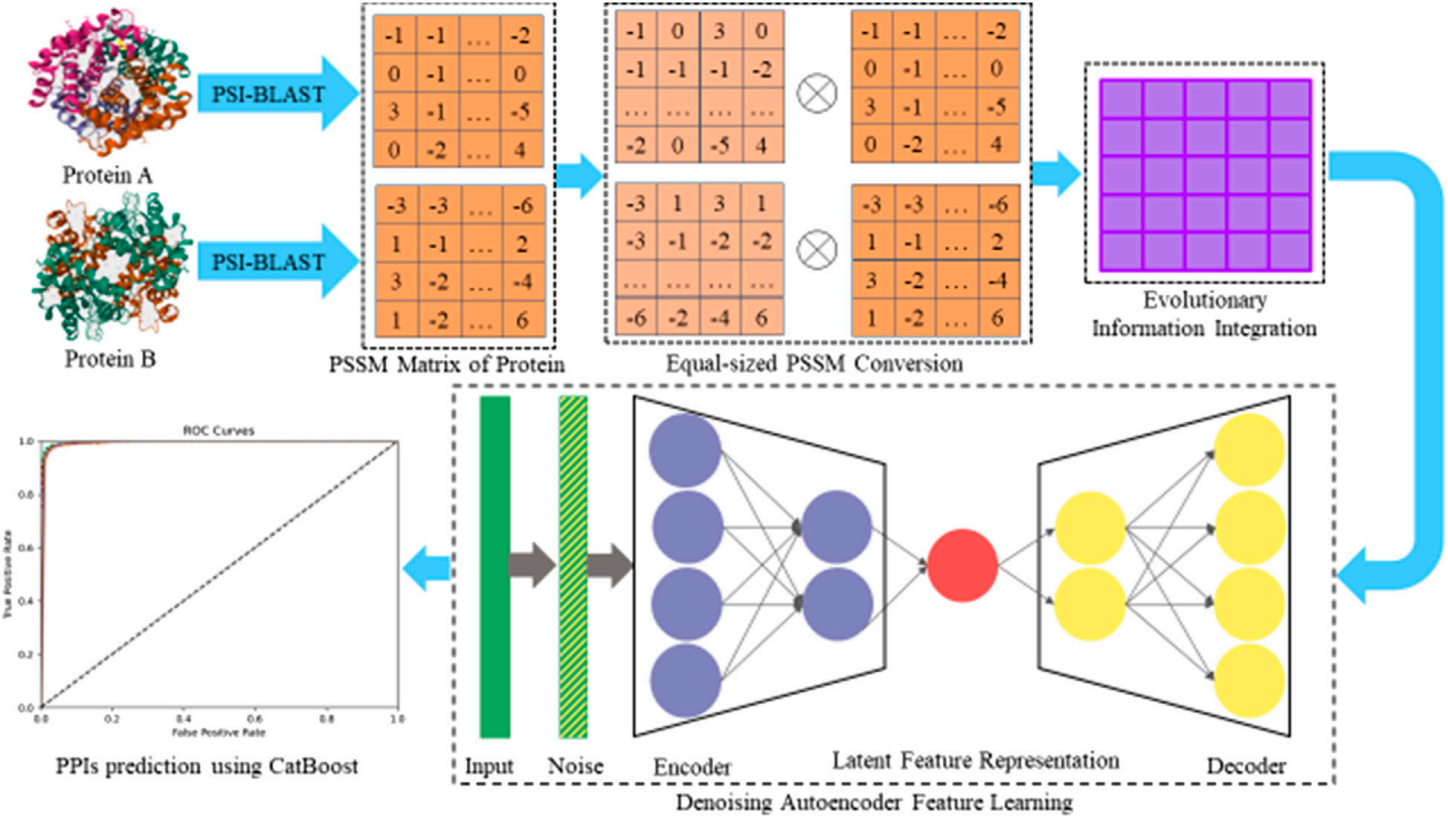
Flowchart of the DAEPPI algorithm.

Here, TP (true positive) refers to true interacting pairs that are predicted to interact by the DAEPPI model, TN (true negative) refers to true non-interacting pairs that are predicted to have no interaction by the DAEPPI model, FP (false positive) refers to pairs that are predicted to interact by the DAEPPI model but do not actually have an interaction, and FN (false negative) refers to pairs that are predicted to be non-interacting by the DAEPPI model but are actually interacting pairs. Furthermore, we calculated the receiver operating characteristic (ROC) curve and the area under the ROC curve (AUC) (Li et al., 2022a) of DAEPPI on two benchmark PPI datasets to further evaluate the stability of DAEPPI. The flowchart of the DAEPPI model is shown in Figure 1.

## 3 Results and discussion

### 3.1 Assessment of prediction

**FIGURE 2:**
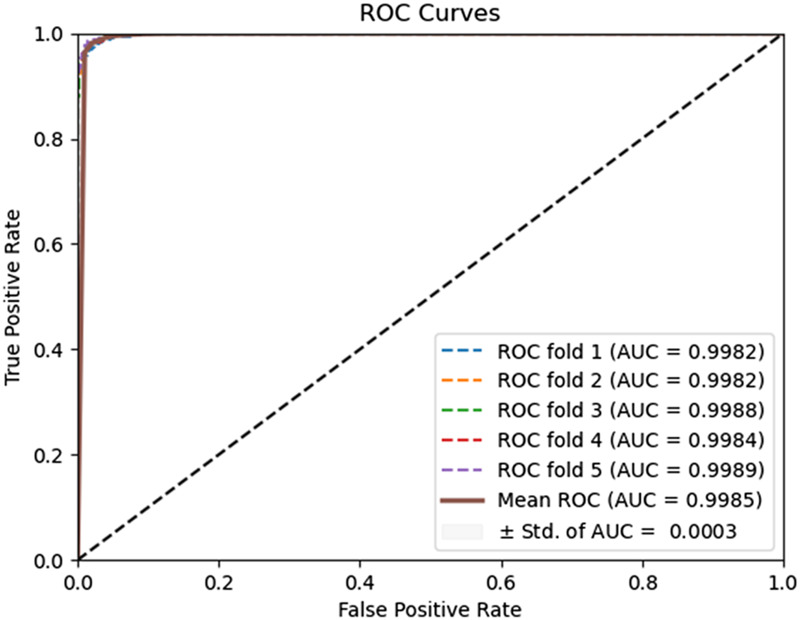
ROC curves of DAEPPI performed on the Yeast dataset.

**FIGURE 3:**
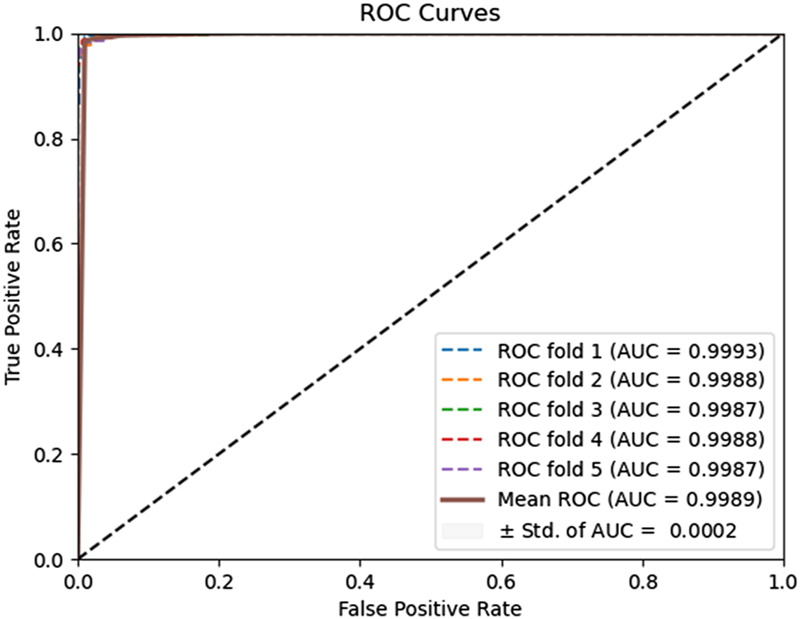
ROC curves of DAEPPI performed on the Human dataset.

To evaluate the predictive performance of our proposed DAEPPI model, which integrates PSSM, DAE, and CatBoost, we conducted extensive assessments on the Yeast and Human datasets using five-fold cross-validation. This method allowed us to partition each dataset into five distinct subsets, ensuring that our model was tested across multiple scenarios for robustness. The results demonstrated that the DAEPPI model achieved impressive performance metrics, as shown in Tables 1–3. For the yeast dataset, we recorded an average ACC of 0.9785, along with PE and SN values of 0.9707 and 0.9870, respectively. The AUC reached 0.9985, indicating a strong predictive capability. Similarly, on the Human dataset, the model maintained high performance, achieving an average ACC of 0.9849, a PE of 0.9929, and an AUC of 0.9989. The standard deviations for these metrics were notably low, reflecting the stability and reliability of our model across different training and testing folds, which further confirms the model’s effectiveness in distinguishing between positive and negative PPIs. The ROC curves of the DAEPPI model on the Yeast and Human datasets are shown in Figures 2, 3. Overall, these results underscore the robustness and accuracy of the DAEPPI model, attributing its success to the effective feature extraction capabilities of the DAE and the powerful classification strength of CatBoost. The integration of evolutionary information through PSSM, combined with advanced machine learning techniques, positions our model as a valuable tool for predicting protein interactions.

**TABLE 1:** Performance of DAEPPI on the Yeast dataset.

Testing set	ACC	PE	SN	MCC	AUC
1	0.9759	0.9708	0.9812	0.9518	0.9982
2	0.9777	0.9668	0.9893	0.9556	0.9982
3	0.9794	0.9669	0.9929	0.9592	0.9988
4	0.9759	0.9692	0.9830	0.9518	0.9984
5	0.9839	0.9796	0.9884	0.9679	0.9989
Average	0.9785 ± 0.0030	0.9707 ± 0.0047	0.9870 ± 0.0043	0.9573 ± 0.0060	0.9985 ± 0.0003

**TABLE 2:** Performance of DAEPPI on the Human dataset.

Testing set	ACC	PE	SN	MCC	AUC
1	0.9859	0.9922	0.9782	0.9718	0.9993
2	0.9816	0.9921	0.9692	0.9634	0.9988
3	0.9859	0.9935	0.9769	0.9719	0.9987
4	0.9871	0.9948	0.9782	0.9743	0.9988
5	0.9841	0.9922	0.9743	0.9682	0.9987
Average	0.9849 ± 0.0019	0.9929 ± 0.0011	0.9754 ± 0.0034	0.9699 ± 0.0038	0.9989 ± 0.0002

**TABLE 3:** Performance comparison of DAEPPI with different PSSM conversion methods.

Datasets	Method	ACC (%)	PE (%)	SN (%)	MCC (%)	AUC (%)
Yeast	Zero padding	78.16 ± 0.38	78.06 ± 0.41	78.35 ± 1.07	56.34 ± 0.77	86.36 ± 0.39
	DAEPPI	97.85 ± 0.30	97.07 ± 0.47	98.70 ± 0.43	95.73 ± 0.60	99.85 ± 0.03
Human	Zero padding	79.99 ± 0.90	80.74 ± 0.92	76.33 ± 1.53	59.90 ± 1.80	88.65 ± 1.02
	DAEPPI	98.49 ± 0.19	99.29 ± 0.11	97.54 ± 0.34	96.99 ± 0.38	99.89 ± 0.02

### 3.2 Comparison with different PSSM transformations

**FIGURE 4:**
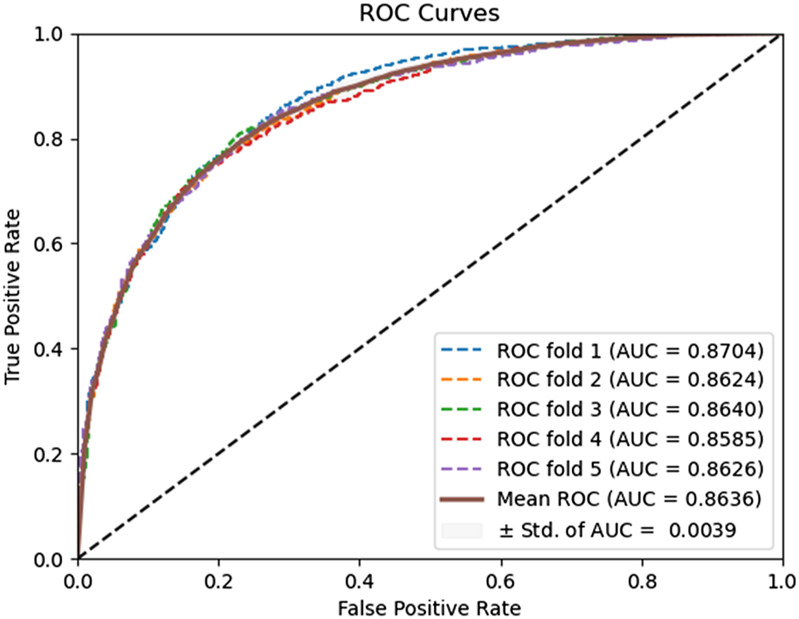
ROC curves performed using the zero padding method on the Yeast dataset.

**FIGURE 5:**
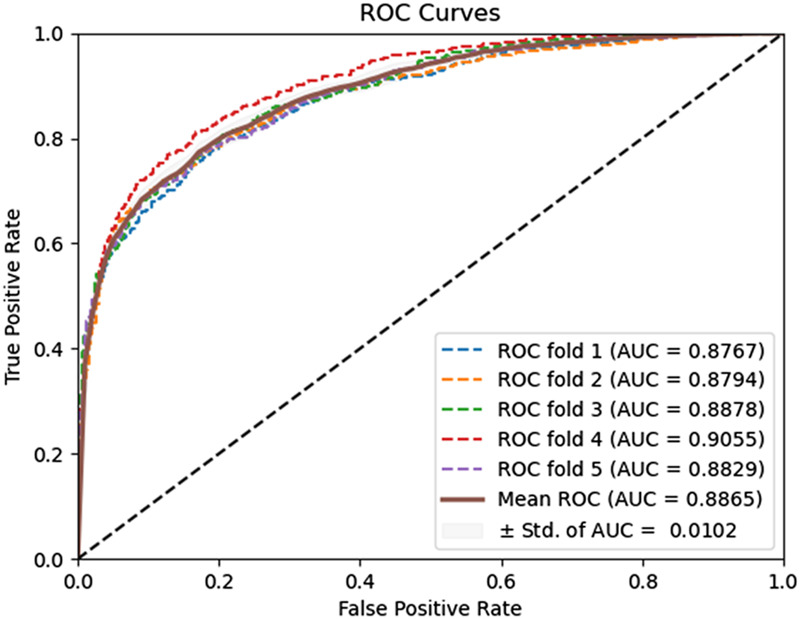
ROC curves performed using the zero padding method on the Human dataset.

In our study, the DAEPPI model employs an equal-sized PSSM transformation, specifically the transposition of PSSM against itself. This approach has demonstrated robust predictive performance on both the yeast and human datasets. To further validate the effectiveness of this equal-sized PSSM transformation, we conducted a comparative analysis with a padded PSSM transformation method. Specifically, we adopt the zero padding method (He and Wang, 2022) to transform the PSSM, i.e., when the length of the protein sequence exceeds the number of amino acids, i.e., 20, we truncate the PSSM to construct a 20×20 PSSM matrix; otherwise, when the length of the protein sequence is lower than the number of amino acids 20, i.e., we need to fill it with zeros. The comparison results are presented in Table 3. The ROC curves of the zero padding method adopted by the model on the Yeast and Human datasets are shown in Figures 4, 5. The experimental results revealed that the equal-size PSSM transformation strategy employed in our model consistently outperforms the zero-padding PSSM transformation method on the two benchmark datasets. Thus, this equal-size transformation enhances the model’s ability to capture the essential features of the protein sequence, leading to improved accuracy and reliability.

### 3.3 Comparison with different feature extraction

**FIGURE 6:**
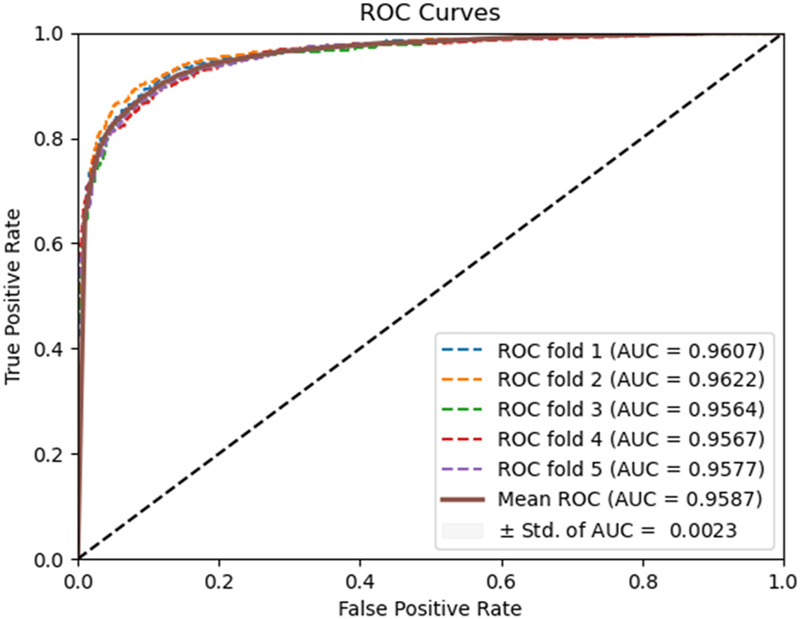
ROC curves performed using the HOG feature extraction method on the Yeast dataset.

**FIGURE 7:**
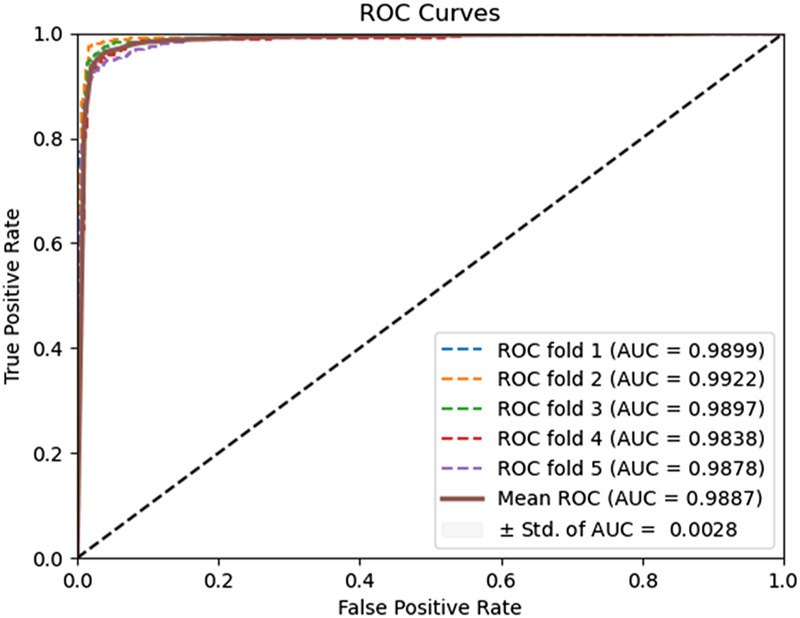
ROC curves performed using the HOG feature extraction method on the Human dataset.

In this experiment, the DAEPPI model utilizes a DAE for in-depth feature extraction from equal-sized PSSM transforms. This approach has shown impressive predictive performance on both yeast and human datasets. To assess the effectiveness of DAE in feature extraction, we compared our model with different feature extraction methods on the same datasets. Specifically, we employed histogram of oriented gradient (HOG) (Admass et al., 2024) for feature extraction from the equal-sized PSSM, while still utilizing CatBoost for predicting PPIs. HOG is a feature descriptor that captures the distribution of gradients in localized portions of an image, making it effective for edge detection and object recognition. The prediction performance of the HOG-based feature extraction model on the benchmark dataset is shown in Table 4. It achieves an average ACC and AUC of 89.22% and 95.87%, respectively, on the Yeast dataset, while attaining 95.31% and 98.87% on the Human dataset, respectively. The ROC curves of the HOG-based feature extraction method on the Yeast and Human datasets are shown in Figures 6, 7. These comparisons suggest that the HOG-based feature extraction model’s performance in capturing intricate evolutionary information in biological data is notably inferior to that of the DAE approach employed in the model. In conclusion, the comparative analysis highlights the superior effectiveness of the DAE-based feature extraction approach in our DAEPPI model. This reinforces the importance of selecting appropriate feature extraction techniques in enhancing the model performance for predicting protein interactions.

**TABLE 4:** Performance comparison of DAEPPI with different feature extraction methods.

Datasets	Method	ACC (%)	PE (%)	SN (%)	MCC (%)	AUC (%)
Yeast	HOG feature	89.22 ± 0.68	90.68 ± 0.82	87.43 ± 1.02	78.50 ± 1.36	95.87 ± 0.23
	DAEPPI	97.85 ± 0.30	97.07 ± 0.47	98.70 ± 0.43	95.73 ± 0.60	99.85 ± 0.03
Human	HOG feature	95.31 ± 0.56	97.95 ± 0.44	92.10 ± 0.85	90.72 ± 1.12	98.87 ± 0.28
	DAEPPI	98.49 ± 0.19	99.29 ± 0.11	97.54 ± 0.34	96.99 ± 0.38	99.89 ± 0.02

### 3.4 Comparison with different classifiers

In our DAEPPI model, we have leveraged CatBoost to predict the intricate features extracted by the DAE, resulting in enhanced performance in predicting PPIs on two benchmark datasets. To validate the effectiveness of CatBoost as our chosen classifier, we conducted comparative experiments on the Yeast dataset, substituting CatBoost with several other classification algorithms. Specifically, we replaced CatBoost with naive Bayes (NB), linear discriminant analysis (LDA), support vector machine (SVM), decision tree (DT), and k-nearest neighbors (KNN), while maintaining all other parameters constant. Our comparison results are shown in Table 5. As can be seen, the DAEPPI model with CatBoost outperformed all other classifiers on the Yeast dataset, demonstrating superior predictive accuracy and reliability for PPIs. This highlights the significant advantage of using CatBoost within our framework, reinforcing its effectiveness over traditional classifiers such as NB, LDA, SVM, DT, and KNN in the context of PPI prediction. In summary, the findings from this analysis validate the choice of CatBoost as a classifier in the DAEPPI model, showcasing its capability to yield better performance in predicting protein interactions compared to other established algorithms.

**TABLE 5:** Performance comparison of DAEPPI with different classifiers on the Yeast dataset.

Models	ACC	PE	SN	MCC	AUC
NB	0.8737 ± 0.0011	0.9913 ± 0.0195	0.7566 ± 0.1368	0.7754 ± 0.1013	0.9946 ± 0.0011
LDA	0.7641 ± 0.0042	0.7256 ± 0.0042	0.8495 ± 0.0066	0.5361 ± 0.0085	0.8309 ± 0.0055
SVM	0.9573 ± 0.0064	0.9385 ± 0.0319	0.9807 ± 0.0372	0.9174 ± 0.0119	0.9984 ± 0.0003
DT	0.9649 ± 0.0039	0.9583 ± 0.0056	0.9721 ± 0.0083	0.9299 ± 0.0078	0.9875 ± 0.0046
KNN	0.9663 ± 0.0038	0.9708 ± 0.0069	0.9616 ± 0.0038	0.9327 ± 0.0076	0.9970 ± 0.0004
CatBoost	0.9785 ± 0.0030	0.9707 ± 0.0047	0.9870 ± 0.0043	0.9573 ± 0.0060	0.9985 ± 0.0003

### 3.5 Comparison with other methods

The DAEPPI model leverages a DAE to extract evolutionary features from the PSSM of protein sequences, combined with CatBoost to enhance the prediction of PPIs. To validate the effectiveness of our model, we conducted a comparative analysis with existing methods on both the Yeast and Human datasets, employing five-fold cross-validation for consistency. Numerous computational methods have been developed for PPI detection, particularly utilizing machine learning algorithms that have garnered significant attention. In this section, we present a comparison between our proposed method and several established models to assess their predictive capabilities. Tables 6, 7 summarize the results obtained from different methods on the Yeast and Human datasets, respectively. From Table 6, it is evident that the accuracy of the existing methods ranges from 90.07% to 96.03%, with sensitivity values between 88.82% and 93.51%, and MCC spanning from 82.10% to 91.83%. In contrast, our DAEPPI model achieved impressive metrics, with an average accuracy of 97.85%, an average sensitivity of 98.70%, and an average MCC of 95.73% on the Yeast dataset. Similarly, the results on the Human dataset, detailed in Table 7, reflect the superiority of our method. The DAEPPI model attained an average accuracy of 98.49%, a precision of 99.29%, and an MCC of 96.99%. This indicates a significant improvement over the existing models. In conclusion, the comparative analysis demonstrates that the DAEPPI model is a robust and reliable approach for predicting PPIs. Its ability to effectively capture evolutionary features through DAE and utilize CatBoost for prediction contributes to its outstanding performance on both datasets, establishing it as a leading method in the field of PPI detection.

**TABLE 6:** Comparative results of other methods on the Yeast dataset.

Models	ACC	PE	SN	MCC
OLPP-RoF (Li et al., 2021b)	0.9007 ± 0.0060	0.9024 ± 0.0056	0.8983 ± 0.0141	0.8210 ± 0.0097
DeepFE-PPI (Yao et al., 2019)	0.9478 ± 0.0061	0.9645 ± 0.0087	0.9299 ± 0.0066	0.8962 ± 0.0123
WSRC (Huang et al., 2016)	0.9250 ± 0.0059	0.9587 ± 0.0089	0.8882 ± 0.0098	0.8609 ± 0.0102
DeepPPI (Du et al., 2017)	0.9443 ± 0.0030	0.9665 ± 0.0059	0.9206 ± 0.0036	0.8897 ± 0.0062
MatFLDA_RFs (Li et al., 2022b)	0.9503 ± 0.0025	0.9914 ± 0.0026	0.9084 ± 0.0047	0.9052 ± 0.0045
MARPPI (Li et al., 2023)	0.9603 ± 0.0076	0.9812 ± 0.0098	0.9351 ± 0.0122	0.9183 ± 0.0132
DAEPPI	0.9785 ± 0.0030	0.9707 ± 0.0047	0.9870 ± 0.0043	0.9573 ± 0.0060

**TABLE 7:** Comparative results of other methods on the Human dataset.

Models	ACC	PE	SN	MCC
OLPP-RoF (Li et al., 2021b)	0.9609 ± 0.0024	0.9656 ± 0.0036	0.9520 ± 0.0034	0.9247 ± 0.0046
WSRC (Huang et al., 2016)	0.9554 ± 0.0032	0.9895 ± 0.0025	0.9165 ± 0.0074	0.9141 ± 0.0058
RPEC (Song et al., 2018)	0.9659 ± 0.0124	0.9618 ± 0.0117	0.9672 ± 0.0141	0.9318 ± 0.0249
LPQ-RoF (Wong et al., 2015)	0.9796 ± 0.0022	0.9835 ± 0.0061	0.9732 ± 0.0073	0.9600 ± 0.0040
GWORVM (An et al., 2019)	0.9456 ± 0.0052	0.9308 ± 0.0109	0.9555 ± 0.0091	0.8951 ± 0.0114
GSRVM (An et al., 2019)	0.9215 ± 0.0120	0.9108 ± 0.0079	0.9178 ± 0.0152	0.8545 ± 0.0235
GARVM (An et al., 2019)	0.9303 ± 0.0088	0.9473 ± 0.0213	0.9059 ± 0.0120	0.8612 ± 0.0145
PSORVM (An et al., 2019)	0.9350 ± 0.0090	0.9640 ± 0.0138	0.9191 ± 0.0112	0.8802 ± 0.0103
SIFT-WELM (Li et al., 2020)	0.9760 ± 0.0057	0.9622 ± 0.0106	0.9894 ± 0.0029	0.9523 ± 0.0112
DAEPPI	0.9849 ± 0.0019	0.9929 ± 0.0011	0.9754 ± 0.0034	0.9699 ± 0.0038

### 3.6 Case studies

CVDs are a leading cause of morbidity and mortality worldwide, characterized by a range of disorders affecting the heart and blood vessels. The complexity of these diseases necessitates a deeper understanding of the molecular interactions that underpin their development and progression (Nasirian and Menichetti, 2023). Previous studies have increasingly highlighted the critical role of PPIs in cardiovascular health, showcasing how alterations in these interactions can contribute to various cardiovascular conditions (Singh et al., 2019). Numerous studies have identified various molecules associated with diseases, including non-coding RNAs (Wang et al., 2023) and protein molecules. In particular, these studies have revealed a strong connection between specific PPIs and cardiovascular disease. For example, interactions involving proteins such as VEGF and its receptors have been implicated in angiogenesis (Li Q. et al., 2021), while proteins involved in lipid metabolism, such as apolipoproteins, are crucial for maintaining vascular health (Mehta and Shapiro, 2022). These insights underscore the importance of exploring the intricate network of PPIs to identify potential biomarkers and therapeutic targets for CVDs.

To further investigate the relevance of the DAEPPI model’s predictions in the context of CVDs, we conducted case study experiments in this section. Our approach involved training the DAEPPI model on the Yeast dataset and subsequently utilizing it to predict PPIs within the Human dataset. The results of the case studies are shown in Table 8, where it can be observed that 11 of the top 15 PPIs predicted by DAEPPI to be associated with CVD have been confirmed by biological experiments. The results revealed several significant PPIs that are closely associated with CVDs, indicating a promising avenue for further research. Through this case study, we aim to demonstrate not only the predictive power of the DAEPPI model but also its potential utility in uncovering critical biological insights that may lead to advancements in CVD prevention and treatment.

**TABLE 8:** Top 15 PPIs predicted by DAEPPI to be related to cardiovascular diseases.

Rank	Protein a	Protein B	Related cardiovascular disease	Evidence
1	NP_612384.1	NP_055105.2	Cardiac hypertrophy	28,746,924
2	NP_002855.1	AAA50404.1	Cardiovascular risk	37,762,835
3	NP_004636.1	NP_004851.1	Cardiac arrhythmogenesis	29,101,288
4	NP_478126.1	NP_002784.1	Cardiovascular disease	28,245,982
5	NP_066921.2	NP_001014797.1	Coronary arterial smooth muscle cells	32,147,517
6	NP_001886.1	NP_932070.1	Cardiac hypertrophy	34,211,403
7	NP_892117.1	NP_006618.1	Chronic vascular inflammation	33,178,683
8	NP_006752.1	NP_068660.1	Cardiovascular disease	Unconfirmed
9	NP_005569.1	NP_542159.2	Heart disease	23,650,592
10	NP_001005.1	NP_008998.1	Cardiovascular disease	Unconfirmed
11	AAH32474.1	NP_055109.1	Cardiovascular disease	Unconfirmed
12	NP_003173.1	NP_060541.3	Plasma levels	10.22391/fppc.779,394
13	NP_006592.3	NP_001017963.1	Cardiovascular disease	Unconfirmed
14	NP_000466.2	NP_004227.1	Cardiovascular disease	31,195,722
15	NP_060848.2	NP_733779.1	Post-myocardial infarction cardiac fibrosis	38,615,011

## 4 Conclusion

Machine learning algorithms have become indispensable in the field of proteomics, particularly for predicting PPIs. These computational methods not only enhance accuracy but also streamline the analysis process, saving both time and resources. In this study, we have introduced the DAEPPI model, which leverages a DAE to learn deep evolutionary features from protein sequences represented in PSSM. By integrating these features with the CatBoost algorithm, our model significantly improves the prediction of PPIs. Our extensive experiments demonstrated the effectiveness of the DAEPPI model, showing robust predictive performance on both Yeast and Human datasets. The results indicate that our method consistently outperforms existing techniques, confirming its reliability and accuracy in PPI prediction. Additionally, we conducted comparative experiments and case studies that further validated the DAEPPI model’s effectiveness. Notably, our analysis identified several human PPIs that are closely associated with CVDs, suggesting that the DAEPPI model can not only predict interactions but also uncover potential links to significant health conditions. In conclusion, the DAEPPI model combines deep learning with effective classification techniques to achieve reliable results. We believe that this model will serve as a valuable tool for researchers aiming to explore the intricate networks of protein interactions and their implications in various biological processes.

## Data Availability

The datasets can be obtained from the corresponding author upon reasonable request.
